# Efeitos do Treinamento Intervalado de Alta Intensidade e do Treinamento Contínuo na Capacidade de Exercício, Variabilidade da Frequência Cardíaca e em Corações Isolados em Ratos Diabéticos

**DOI:** 10.36660/abc.20220396

**Published:** 2022-12-20

**Authors:** Eduardo Gomes de Souza, João Victor Capelli Peixoto, Claucio Rank, Ricardo Rasmussen Petterle, Rosalvo Tadeu Hochmuller Fogaça, Beata Maria Wolska, Fernando Augusto Lavezzo Dias

**Affiliations:** 1 Universidade Federal do Paraná Departamento de Fisiologia Curitiba PR Brasil Universidade Federal do Paraná – Departamento de Fisiologia , Curitiba , PR – Brasil; 2 Universidade Federal do Paraná Departamento de Medicina Integrada Curitiba PR Brasil Universidade Federal do Paraná – Departamento de Medicina Integrada , Curitiba , PR – Brasil; 3 University of Illinois at Chicago Chicago Illinois EUA University of Illinois at Chicago – Medicine, Physiology and Biophysics, Chicago , Illinois – EUA

**Keywords:** Exercício, Esforço Físico, Diabetes Mellitus, Ratos, Frequência Cardíaca

## Abstract

**Fundamento:**

O treinamento intervalado de alta intensidade (HIIT) tem sido sugerido como alternativa ao treinamento contínuo (TC) em indivíduos com diabetes mellitus (DM) devido à sua curta duração e potencial para melhorar a adesão ao exercício. No entanto, dados sobre seu impacto sobre a variabilidade da frequência cardíaca (VFC) são escassos.

**Objetivos:**

Avaliar e comparar os efeitos do HIIT e TC sobre a capacidade no exercício, VFC e corações isolados em ratos diabéticos.

**Métodos:**

Animais diabéticos (estreptozotocina intravenosa, 45 mg.kg ^-1^ ) e controles (C) realizaram 20 sessões de TC (5 dias/semana, 50 min, por quatro semanas) em esteira (70% da capacidade máxima de exercício) ou HIIT (ciclos de 1:1min a 50% e 90% da capacidade máxima de exercício). A VFC foi avaliada por eletrocardiograma contínuo, e a função cardíaca foi avaliada em corações isolados perfundidos. Para a análise dos dados, utilizamos a matriz do modelo linear generalizado de covariância multivariada ou o teste one-way ANOVA seguido pelo teste de Tukey, considerando um valor de p<0,05 como significativo.

**Resultados:**

A capacidade de exercício (m/min) foi maior no grupo submetido ao HIIT [DM-HIIT: 36,5 (IIQ 30,0-41,3); C-HIIT: 41,5 (37,8-44,5), ambos n=10) em comparação ao grupo submetido ao TC [DM-TC: 29,0 (23,8-33,0); C-TC: 32,0 (29,5-37,0), ambos n=10) (p<0,001). A frequência cardíaca (bpm) foi mais baixa no grupo DM em comparação aos controles (p<0,001) tanto *in vivo* (DM-HIIT: 348±51, C-HIIT:441±66, DM-TC:361±70, C-TC:437±38) como nos corações isolados. Não houve diferenças na VFC entre os grupos. Os valores máximos e mínimos de dP/dt foram reduzidos no DM, com exceção da +dP/dt no grupo DM-HIIT vs. C-HIIT (diferença média: 595,5±250,3, p=0,190).

**Conclusão:**

O HIIT de curto prazo promoveu melhora superior no desempenho no exercício em comparação ao TC, sem causar mudanças significativas na variabilidade da frequência cardíaca.

## Introdução

Diabetes mellitus (DM) é um fator de risco para doenças cardiovasculares e está associado com mortalidade por todas as causas e mortalidade cardiovascular mais elevadas. ^1^ Entre as complicações conhecidas do DM encontra-se a neuropatia. ^2^

A neuropatia autonômica cardíaca (NAC), frequentemente observada em indivíduos com DM, ^3^ afeta as fibras autonômicas que inervam o coração e os vasos sanguíneos. ^4^ Um comprometimento da função nervosa causa alterações fisiológicas, tais como aumento da frequência cardíaca (FC) e redução na variabilidade da frequência cardíaca (VFC) em repouso nesses indivíduos. ^5^ Além dos pacientes com DM, reduções na VFC também são relatadas em doenças relacionadas ao estilo de vida sedentário, tais como hipertensão, ^6^ obesidade, ^7^ e infarto do miocárdio. ^8^ Assim, a prática regular de exercícios físicos tornou-se uma importante ferramenta na promoção da saúde. ^9^

Animais diabéticos apresentam capacidade física reduzida em comparação a controles. ^10 , 11^ No coração, essa condição reflete negativamente na função sistólica ^10 - 12^ e diastólica ^10^ e está associada à sobrecarga cardíaca. ^10^ O treinamento físico restaura a capacidade funcional, com melhora na disfunção cardíaca, em animais diabéticos. ^10 , 11 , 13^ Contudo, a magnitude da melhora depende da intensidade do exercício e pode ser dependente da modalidade do treinamento. ^13 , 14^

O HIIT (Treino Intervalado de Alta Intensidade, ou *high-intensity interval training* em inglês) tem sido considerado uma maneira eficaz de aumentar a regularidade na prática de atividade física devido à menor duração ^15^ e melhores respostas adaptativas em comparação ao treinamento contínuo (TC). Por isso, o HIIT tem sido sugerido para indivíduos diabéticos, ^16 - 18^ mesmo sem um conhecimento aprofundado acerca das repercussões fisiológica no controle autonômico cardíaco nessa população. ^19^ Evidência clara da segurança do HIIT, bem como dos efeitos benéficos sobre o sistema, se faz necessária para a recomendação do HIIT a pacientes diabéticos.

Nossa hipótese é a de que, em comparação ao TC, o programa de HIIT melhore a capacidade de exercício, mas exerça efeitos diferentes sobre a VFC e a contratilidade cardíaca em um modelo de DM induzido por estreptozotocina (STZ). Assim, o objetivo deste estudo foi avaliar, em ratos com DM induzido por STZ, os efeitos de um protocolo de curto prazo de HIIT ou de TC sobre a capacidade de exercício, a VFC avaliada por eletrocardiograma (ECG) e sobre a função cardíaca em coração isolado perfundido.

## Métodos

### Animais e delineamento experimental

Sessenta ratos Wistar machos, com peso entre 250 e 300 g foram mantidos em gaiolas sob condições controladas de temperatura e ciclo claro-escuro de 12 horas, com livre acesso a alimentos e água, e separados aleatoriamente em grupo controle (C, n=30) e grupo DM (DM, n=30). Esses grupos foram subdivididos em animais controles não treinados (C-NT, n=10), animais diabéticos não treinados (DM-NT, n=10), animais controles em TC (C-TC, n=10), animais diabéticos em TC (DM-TC, n=10), animais controles em HIIT (C-HIIT, n=10) e animais diabéticos em HIIT (DM-HIIT, n=10). Todos os protocolos experimentais usados no estudo foram aprovados pela Comissão de Ética no Uso de Animais do Setor de Ciências Biológicas da Universidade Federal do Paraná (CEUA-BIO – UFPR) (número de aprovação: AEEC-866), e conduzidos de acordo com as diretrizes do Conselho Nacional de Controle de Experimentação Animal (CONCEA).

O tamanho da amostra inicial foi calculado usando o GPower 3.1, com base na porcentagem de aumento da capacidade de exercício observado em estudos anteriores. Foram considerados seis grupos independentes com mesmo número (n) de animais, tamanho do efeito de 0,6, poder de 0,85, e alfa de 0,05, resultando em um tamanho amostral de 48 animais (oito por grupo).

**Figura 1:**
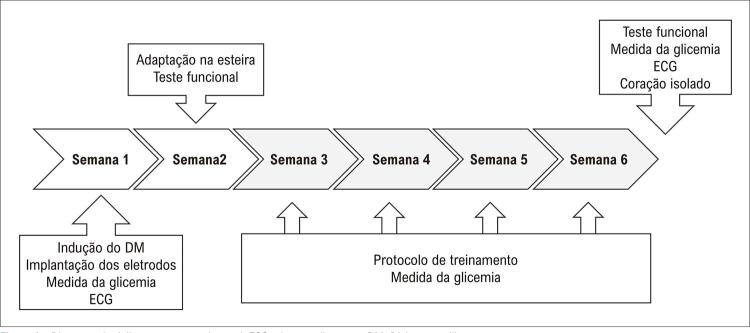
Diagrama do delineamento experimental; ECG: eletrocardiograma; DM: Diabetes mellitus.

O delineamento experimental está representado na Figura 1 e descrito detalhadamente a seguir.

### Implantação dos eletrodos e indução de DM

Após 14 horas de jejum, os animais foram anestesiados com cetamina (80 mg kg ^-1^ , Ketalex, Dechra, Brasil) e xilazina (10 mg kg ^-1^ , Xilazin, Syntec, Brasil) para o procedimento. Quatro eletrodos (aço inoxidável, 0,5 mm) subcutâneos foram implantados posteriormente aos membros inferiores e superiores, conforme descrito por Marques Neto et al., ^20^ para o monitoramento por ECG. O DM foi induzido por injeção intravenosa (veia peniana) de STZ (45 mg.kg ^-1^ , Sigma-Aldrich, Alemanha) solubilizada com 0,01M de tampão citrato, pH 4,5. ^21^ No grupo controle, somente tampão citrato foi injetado. A glicemia capilar (precedida de seis horas de jejum) foi medida a partir de amostra colhida da cauda dos animais, por meio de um glicosímetro digital (Accu-check Performa, Roche Diagnostic, Alemanha). As medidas foram realizadas antes da injeção de STZ, sete dias após a injeção para confirmação do estado hiperglicêmico (glicemia de jejum >250 mg dL ^-1^ ), bem como no início, no 15º dia, e no final do protocolo de treinamento.

### Monitoramento por ECG e cálculo da VFC

Os animais foram mantidos em gaiolas individuais e o ECG foi registrado 24 horas após a confirmação do DM, antes do teste de desempenho (descrito abaixo), e repetido uma vez por semana durante as quatro semanas do protocolo de exercício. As medidas foram realizadas por uma hora, sempre pela manhã, para evitar a influência do ciclo circadiano, ^22^ sem restrição ou anestesia (cabos foram conectados aos fios implantados, mas os animais eram capazes de se moverem dentro das gaiolas). Os experimentos foram realizados em uma sala reservada e silenciosa, a uma temperatura de 20 ^o^ C.

No ECG, imagens do plano frontal foram adquiridas usando o sistema PowerLab, modelo 26T (AD Instrument, Austrália) e analisadas usando o programa Lab Chart versão 7.0 (AD Instrument, Austrália). Para a VFC, os dados obtidos da derivação DII foram processados no LabChart e em seguida transferidos para o Kubius HRV (2.0 MATLAB, MathWorks, Inc., Finlândia) para análises.

A VFC foi medida a partir da aquisição de intervalos R-R em resolução de 1 milissegundo. Os dados foram analisados nos domínios do tempo e da frequência, usando a área de maior estabilidade nos intervalos R-R, correspondendo a 10 minutos de registros. Para os parâmetros do domínio tempo, calculamos o desvio padrão dos intervalos RR normais (SDNN), a raiz quadrada da média dos quadrados das diferenças entre os intervalos RR (RR SDNN), e a FC. Os domínios da frequência foram analisados pela transformada rápida de Fourier após subtrair a tendência linear utilizando-se filtros automáticos. Depois calculamos a baixa frequência (LF) (0,20 a 0,75 Hz), a alta frequência (HF) (0,75 a 3Hz) e a razão LF/HF. Ainda, foi realizada a análise não linear para SD1 (desvio padrão da variabilidade instantânea RR) e SD2 (desvio padrão da variabilidade contínua ou de longo prazo da FC). ^23^

### Teste de desempenho e protocolo de treinamento

O treinamento do exercício foi realizado em uma esteira motorizada desenhada para roedores (Insight Equipamentos Ltda, Brasil), cuja velocidade é controlada por um computador. Tanto o teste de desempenho como o protocolo de treinamento foram adaptados de Pereira et al. ^24^

Vinte e quatro horas após a confirmação do DM, os animais começaram a ser adaptados a correrem sobre a esteira. A adaptação foi conduzida em cinco dias consecutivos, 50 minutos por dia, sem inclinação. No primeiro dia, os animais caminharam a 5m/min. A velocidade foi aumentada a 1m/min por dia. No sexto dia, os animais foram submetidos a um protocolo de exaustão, com objetivo de identificar a velocidade máxima de corrida de cada animal. O teste consistiu em exercício graduado (sem inclinação), começando a 5m/min, com incrementos de 1m/min a cada minuto, até a velocidade máxima de corrida alcançada por cada rato (exaustão). Quando o animal não pôde manter a velocidade por um minuto, a velocidade anterior em que os animais conseguiram correr foi considerara como a velocidade máxima atingida.

As sessões de treinamento começaram 48 horas após o teste, e foram realizadas cinco vezes por semana (de segunda-feira a sexta-feira) por quatro semanas, 50 minutos por sessão. Todas as sessões iniciaram com um aquecimento de cinco minutos e terminaram com um resfriamento a 5m/min por cinco minutos. Os grupos de TC correram a 70% da capacidade máxima individual por 40 minutos. Os grupos de HIIT correram a 90% da capacidade máxima individual por um minuto, intercalado por um minuto a 50% por 40 minutos. Os grupos não treinados foram mantidos sobre a esteira por 50 minutos sem se exercitarem. Após 48 horas da última sessão de treinamento, os animais foram novamente submetidos ao teste de desempenho descrito acima.

### Função cardíaca no coração isolado perfundido

A função cardíaca foi avaliada em corações isolados perfundidos como descrito anteriormente. ^25^ Em resumo, os animais foram pesados, anestesiados com injeção intraperitoneal de cetamina (80 mg kg ^-1^ ) e xilazina (20 mg kg ^-1^ ), e submetidos à eutanásia por exsanguinação. O peito foi aberto, o coração coletado e rapidamente transferido para um sistema de perfusão Langendorff, permitindo perfusão imediata com solução de Krebs-Henseleit. Essa solução apresentava a seguinte composição (em mM): NaCl = 118,5; KCl = 5, CaCl _2_ = 1,5; KH _2_ PO _4_ = 2,0; MgSO _4_ = 1,2; NaHCO _3_ = 26 e glicose = 10, mantida a pH 7,4, e gaseificada com uma mistura de O _2_ (95%) e CO _2_ (5%) a 37°C e pressão de perfusão constante de 60 mmHg. Para a medida da pressão ventricular esquerda, uma porção do átrio esquerdo foi removido, e um balão conectado a um transdutor de pressão (WPI-rBPI, USA) foi inserido no ventrículo esquerdo através da valva mitral. O volume do balão foi gradualmente ajustado para obter a pressão máxima desenvolvida em batimentos cardíacos espontâneos. Os dados foram obtidos pelo sistema de aquisição PowerLab 26T e analisados pelo programa Lab Chart versão 7.3.7 (AD Instruments, Austrália).

### Análise estatística

Os resultados foram expressos como média e desvio padrão ou mediana e intervalo interquartil para dados não paramétricos. Para análises dos dados, usamos a matriz do modelo linear generalizado de covariância multivariada ^26^ por meio do pacote McGLM ^27^ e correção apropriada para a distribuição dos dados usando o teste Tweedie. Utilizamos o teste de Shapiro-Wilk para avaliar se as variáveis seguiam uma distribuição normal. Para dados do coração isolado, uma vez que possuíamos apenas um ponto de medida, usamos o teste one-way ANOVA e o teste de Tukey para comparações múltiplas.

Para análise e plotagem dos dados, usamos o programa R (the R foundation, https://www.r-project.org/), ou o programa Graph Pad Prism 8 (Graph Pad Software, San Diego, California, USA). O nível de significância adotado foi p < 0,05.

## Resultados

### Peso corporal e capacidade de exercício

Peso corporal, glicemia e capacidade no exercício antes e após o protocolo de exercício nos animais controles e diabéticos estão apresentados na Tabela 1 . Não foram observadas diferenças de peso corporal entre os grupos (0,877) no basal. O peso diminuiu no grupo DM (estimativa: -19,1; p<0,001) e aumentou no grupo controle (estimativa: 66,57, p<0,001) durante o acompanhamento. O peso corporal foi significativamente mais baixo em animais diabéticos em comparação aos controles após o período de exercício (p<0,001). Não houve efeito da modalidade do exercício sobre a alteração de peso corporal após o período de acompanhamento (p=0,91 ao se comparar as modalidades de exercício).

**Tabela 1:** Peso corporal, glicemia, e capacidade no exercício em animais diabéticos e controles antes e após o protocolo de exercício

	C-NT (n=10)	DM-NT (n=10)	C-CT (n=10)	DM-CT (n=10)	C-HIIT (n=10)	DM-HIIT (n=10)
**Peso corporal (g)**						
Antes	266,50 ± 8,41	281,00 ± 22,53	261,00 ± 26,23	250,80 ± 16,71	261,10 ± 22,61	259,80± 19,27
Após	342,60 ± 13,18 ^a^	257,40 ± 33,27 ^a,b^	320,30 ± 31,71 ^a^	247,00 ± 44,22 ^a,b^	325,40 ± 16,31 ^a^	229,90 ± 24,28 ^a,b^
**Glicemia(mg/dL)**						
Antes	94,60 ± 10,33	462,90 ± 61,21 ^b^	102,50 ± 3,69	450,00 ± 143,5 ^b^	106,30 ± 10,33	489,60 ± 72,22 ^b^
Após	94,30 ± 5,48	504,20 ± 66,55 ^a,b^	104,10 ± 7,31	542,00 ± 60,60 ^a,b^	106,00 ± 8,97	521,90 ± 64,08 ^a,b^
**Velocidade máxima de corrida (m/min)**						
Antes	27,00 (25,75-29,25)	25,5 (23,00-27,25)	25,00 (24,50-28,00)	24,50 (23,25-25,00)	25,00 (24,50-31,00)	24,50 (20,75-25,00)
Após	25,00(23,75-25,50) ^a^	21,50 (19,00-25,25) ^a^	32,00 (29,50-37,00) ^a,c^	29,00 (23,75-33,00) ^a,c^	41,50 (37,75-44,50) ^a,c,d^	36,50 (30,00-41,25) ^acd^

*Dados expressos em média ± DP ou mediana (intervalo interquartil). a Diferença estatisticamente significativa (p<0,01) em comparação a antes do exercício (basal); b Diferença estatisticamente significativa (p<0,01) em comparação ao controle; c Diferença estatisticamente significativa (p<0,01) em comparação ao grupo não treinado; d Diferença estatisticamente significativa (p<0,01) em comparação ao grupo submetido a treinamento contínuo; C-NT: animais controles não treinados; DM-NT: animais diabéticos não treinados; C-TC animais controles em treinamento contínuo (C-TC, n=10), DM-TC: animais diabéticos em treinamento contínuo; C-HIIT: animais controles em treinamento intervalado de alta intensidade; DM-HIIT: animais diabéticos em treinamento intervalado de alta intensidade*

Os valores de glicemia foram mais altos nos animais diabéticos em comparação aos animais controles no basal (estimativa: 366,4g/dL; p<0,001) e no seguimento (estimativa: 421,2mg/dL; p<0,001). A glicemia aumentou ainda mais durante o seguimento no grupo DM mas não no grupo C (p=0,982). Não houve efeito significativo do exercício sobre os níveis glicêmicos ( Tabela 1 ).

A velocidade máxima de corrida não foi diferente entre os grupos no basal. Animais não treinados demonstraram uma redução significativa na velocidade máxima de corrida no seguimento. Embora ambas as modalidades de exercício tenham melhorado a velocidade máxima de corrida, o HIIT promoveu maior desempenho em comparação ao TC. A média da velocidade máxima de corrida aumentou em 27.3% no C-TC e 21,4% no DM-TC. Os animais submetidos ao treino intervalado tiveram sua capacidade aumentada, em média, em 52,9% no grupo C-HIIT e 57,7% no grupo DM-HIIT. Não houve diferença entre DM e controles na variação da velocidade máxima de corrida promovida pelo protocolo de exercício no seguimento (ausência de significância estatística quando a interação tripla foi avaliada).

### Domínios de tempo e frequência, e medidas não lineares da VFC

Parâmetros da FC e da VFC durante o período experimental estão resumidos na Tabela 2 . Não foram observadas diferenças nos parâmetros de VFC relacionadas à variabilidade global ou ao equilíbrio simpático-vagal entre os grupos antes ou após o protocolo de exercício. No entanto, observou-se uma redução na FC entre os animais com DM e animais controles.

**Tabela 2:** Parâmetros de frequência cardíaca e variabilidade da frequência cardíaca em ratos diabéticos e ratos controles antes e após o protocolo do exercício

	C-NT	DM-NT	C-TC	DM-TC	C-HIIT	DM-HIIT
**Frequência cardíaca (bpm)**						
Antes	404,15 ± 49,33	358,15 ± 41,51 ^b^	405,62 ± 42,30	372,06 ± 50,80 ^b^	411,47 ± 32,37	339,50 ± 50,52 ^b^
Após	405,74 ± 36,48	342,69 ± 43,89 ^b^	436,74 ± 38,21	361,18 ± 70,72 ^b^	441,26 ± 66,60	347,80 ± 50,56 ^b^
**SDNN (ms)**						
Antes	7,20 (5,84-10,14)	10,25 (6,26-15,03)	6,37 (4,51-8,08)	8,33 (5,81-13,61)	10,69 (6,44-11,84)	9,11 (6,91-11,22)
Após	6,20 (5,440-9,00)	11,54 (6,59-13,92)	8,30 (5,90-11,83)	10,49 (7,00-13,91)	8,52 (5,86-12,08)	7,94 (6,14-10,03)
**RMSSD (ms)**						
Antes	3,56 (2,37-3,66)	2,57 (2,15-3,64)	3,13 (2,16-4,05)	3,25 (2,29-3,54)	3,01 (2,56-4,20)	3,13 (2,20-5,72)
Após	3,06 (2,26-4,05)	4,21 (2,41-5,20)	2,91 (2,02-4,41)	3,44 (2,59-4,26)	2,80 (1,89-3,765)	2,67 (1,90-3,64)
**Potência total (ms ^**2**^ )**						
Antes	36,02 (26,69-59,07)	66,97 (16,97-129,4)	32,99 (14,93-59,02)	38,62 (32,32-103,8)	62,89 (31,87-92,58)	58,05 (22,33-81,79)
Após	33,91 (20,56-64,02)	83,56 (34,71-115,4)	65,14 (25,50-138,9)	79,64 (32,93-143,3)	60,77 (17,32-117,6)	42,32 (27,22-98,79)
**LF (ms** ^**2**^ **)**						
Antes	3,63 (1,42-4,98)	1,93 (1,26-5,32)	2,31 (1,13-8,68)	2,20 (1,90-3,76)	2,45 (1,95-11,4)	3,40 (1,29-12,47)
Após	2,62 (1,51-7,91)	5,88 (1,72-13,86)	2,37 (0,71-15,36)	2,78 (1,84-6,57)	3,09 (1,41-7,37)	3,25 (1,65-5,17)
**HF (ms** ^**2**^ **)**						
Antes	5,43 (3,45-6,33)	2,70 (1,16-7,79)	4,01 (1,44-9,31)	5,36 (2,08-7,59)	4,92 (3,06-9,81)	5,49 (1,44-9,31)
Após	4,02 (1,82-8,02)	6,23 (2,91-10,29)	4,03 (1,57-8,40)	4,12 (2,57-6,91)	4,33 (0,99-7,85)	1,87 (1,20-4,81)
**LF/HF**						
Antes	0,79 (0,56-1,04)	0,78 (0,47-1,41)	0,55 (0,44-1,53)	0,74 (0,47-0,98)	0,66 (0,52-1,13)	0,91 (0,65-1,18)
Após	0,78 (0,57-1,44)	0,95 (0,59-1,46)	0,69 (0,40-1,42)	0,77 (0,43-1,04)	0,70 (0,49-1,00)	1,25 (0,79-1,75)
**SD1**						
Antes	2,52 (1,68-2,59)	1,82 (1,52-2,58)	2,21 (1,53-2,86)	2,30 (1,62-2,50)	2,13 (1,81-2,97)	2,46 (1,56-4,04)
Após	2,16 (1,60-2,86)	2,98 (1,71-3,67)	2,06 (1,43-3,12)	2,44 (1,83-3,02)	1,98 (1,34-2,66)	1,89 (1,33-2,57)
**SD2**						
Antes	9,94 (8,08-13,98)	14,29 (8,70-21,05)	8,49 (6,02-11,04)	11,59 (7,97-19,16)	14,80 (8,81-16,52)	12,17 (9,58-15,57)
Após	8,57 (7,42-12,39)	16,04 (9,24-19,41)	11,68 (8,06-16,43)	14,68 (9,58-19,32)	11,77 (8,07-16,91)	10,90 (8,53-14,06)

*Dados apresentados em média DP ou mediana (intervalo interquartil). SDNN: desvio padrão dos intervalos RR normais; RR SDNN: raiz quadrada da média dos quadrados das diferenças entre os intervalos; LF: baixa frequência; HF: alta frequência; SD1: desvio padrão da variabilidade instantânea RR; SD2: desvio padrão da variabilidade contínua ou de longo prazo da frequência cardíaca. ^*b*^ Diferença estatisticamente significativa (p<0,01) em comparação ao controle; N é igual a 10 exceto para os parâmetros de variabilidade da frequência cardíaca no grupo C-TC (n=9). C-NT: animais controles não treinados; DM-NT: animais diabéticos não treinados; C-TC animais controles em treinamento contínuo (C-TC, n=10), DM-TC: animais diabéticos em treinamento contínuo; C-HIIT: animais controles em treinamento intervalado de alta intensidade; DM-HIIT: animais diabéticos em treinamento intervalado de alta intensidade*

### Coração isolado perfundido

**Figura 2:**
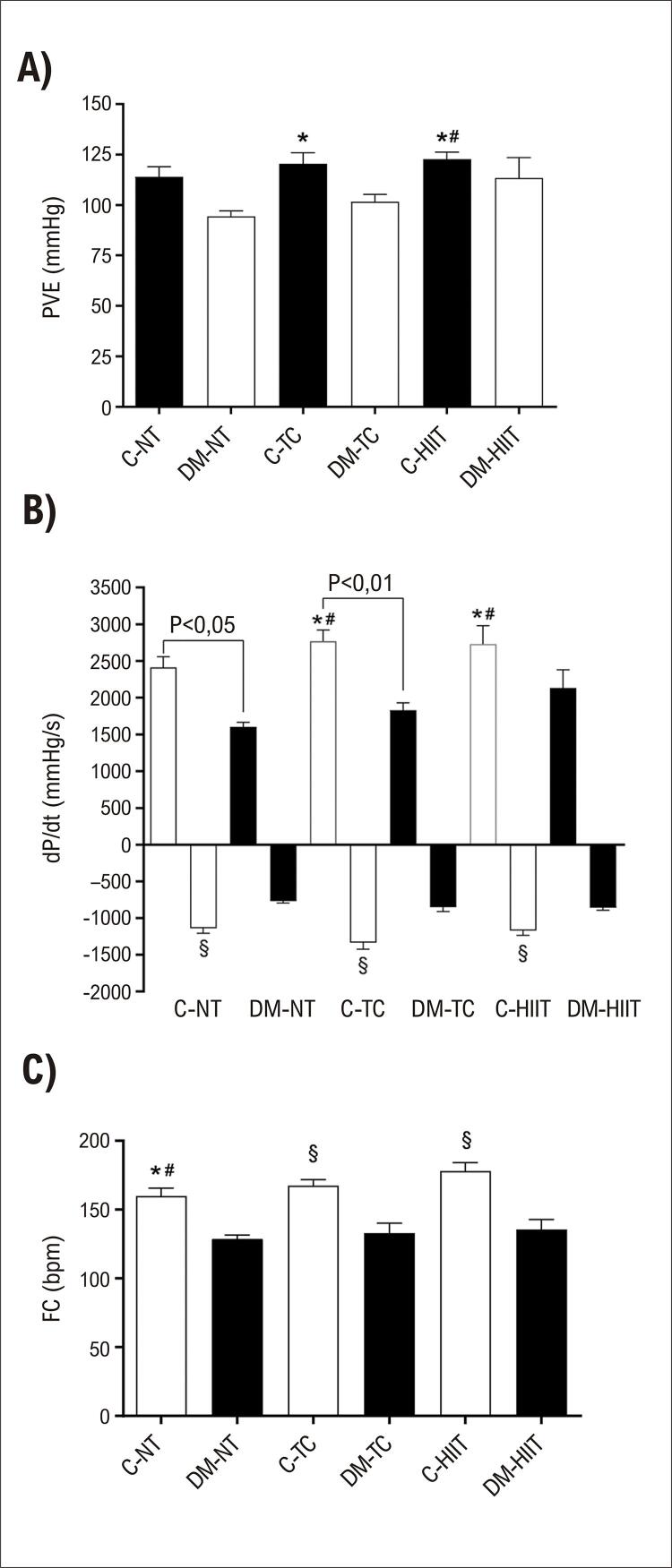
Frequência cardíaca, pressão desenvolvida no ventrículo esquerdo, e taxas máximas do aumento e da queda da pressão ventricular nos corações isolados. A: pressão desenvolvida no ventrículo esquerdo (PVE); B: taxa máxima do aumento (+dP/dt) e da queda (–dP/dt) da pressão ventricular; C: Frequência cardíaca (FC). *diferença significativamente estatística (p<0,01) em comparação a DM-NT, # diferença significativamente estatística (p<0,05) em comparação a DM-CT, § diferença significativamente estatística (p<0,05) em comparação a todos os grupos DM; C-NT: animais controles não treinados; DM-NT: animais diabéticos não treinados; C-TC animais controles em treinamento contínuo (C-TC, n=10), DM-TC: animais diabéticos em treinamento contínuo; C-HIIT: animais controles em treinamento intervalado de alta intensidade; DM-HIIT: animais diabéticos em treinamento intervalado de alta intensidade.

A pressão no ventrículo esquerdo está representada na Figura 2 . Animais controles submetidos ao exercício diferiram-se dos grupos DM-NT, C-HIIT e DM-CT; contudo, não houve diferenças entre animais com DM e controles submetidos às mesmas condições ( *i.e* ., NT ou tipos diferentes de treinamento; p = 0,086 para animais NT, p = 0,061 para TC e p = 0,824 para HIIT).

Diferenças significativas foram observadas em +dP/dt (mmHg/s) nos grupos DM em comparação aos respectivos controles (C-NT vs. DM-NT, diferença média: 812,8 ± 228,5, p = 0,012; C-CT vs. DM-CT, diferença média: 937,5 ± 207,7, p = 0,0008) exceto para HIIT (C-HIIT vs. DM-HIIT, diferença média: 595,5 ± 250,3, p = 0,190) ( Figura 2B ). Valores mais baixos também foram observados para–dP/dt ( Figura 2B ) em todos os grupos DM em comparação aos controles. FC espontânea ( Figura 2C ) reduziu em todos os grupos DM em comparação aos controles (C-NT vs. DM-NT, diferença média: 31,08 ± 8,48; C-CT vs. DM-CT, diferença média: 34,23 ± 7,71; C-HIIT vs. DM-HIIT, diferença média: 42,58 ± 9,29).

## Discussão

O presente estudo comparou, em ratos com DM induzido por STZ, os efeitos de 20 sessões de HIIT ou TC sobre a capacidade de exercício, função cardíaca e VFC. Nossos achados mostraram que ambos os tipos de treinamento melhoraram o desempenho no exercício, com melhores resultados obtidos com HIIT, em magnitudes comparáveis entre animais com DM e controles. Ainda, não houve diferenças na VFC entre controles e animais diabéticos após os protocolos de exercício.

Animais diabéticos submetidos ao treinamento aumentaram a capacidade de exercício ( Tabela 1 ), em contraste com animais sedentários, cuja velocidade máxima de corrida foi significativamente reduzida. No entanto, animais com DM e animais controles submetidos ao HIIT mostraram melhora superior na velocidade máxima de corrida em comparação aos animais submetidos ao TC, embora os níveis glicêmicos não tenham se alterado. A superioridade do treino intervalado sobre o TC havia sido descrita em um modelo de síndrome metabólica envolvendo ratos, em que o VO _2max_ foi superior e associado a uma maior redução na pressão arterial, melhor função endotelial, e ação superior da insulina. ^14^ Em um modelo de diabetes com camundongos, o HIIT reduziu a glicemia de jejum e aumentou a concentração de GLUT4 muscular, contudo, esse protocolo de estudo foi realizado por 10 semanas em comparação a quatro semanas em nosso estudo, o que pode explicar a diferença nos níveis de glicemia. ^28^ Ainda, em humanos, Driller et al., ^29^ mostraram melhora na potência, captação máxima de oxigênio, e limiar de lactado em remadores submetidos a HIIT em comparação ao treino convencional. Em pacientes com diabetes tipo 2, uma revisão sistemática recente descreveu um maior aumento no VO _2max_ no HIIT *versus* TC, sem diferenças significativas no HbA1c ou valores de pressão arterial entre as intervenções, similar a nossos achados. ^30^ A maior melhora na capacidade física encontrada em nosso estudo usando um HIIT de curto prazo está de acordo com respostas adaptativas promovidas em protocolos de HIIT previamente relatadas.

A análise da função cardíaca em corações isolados revelou que não houve diferença estatística na pressão desenvolvida no ventrículo esquerdo em corações com batimentos espontâneos entre os grupos submetidos ao mesmo protocolo de exercício ( Figura 2A ). Contudo, o grupo DM-HIIT mostrou atenuação no distúrbio da razão +dP/dt, o que não foi observado no grupo DM-TC ( Figura 2B ). Não foi observada melhora na função diastólica, representada por -dP/dt, em nenhum dos grupos com DM submetidos aos exercícios ( Figura 2B ). Estudos prévios correlacionaram um aumento na capacidade do exercício com melhora na função sistólica em DM. Sanches et al., ^10^ usaram um protocolo combinado de exercício aeróbico (esteira) e treinamento resistido (escada) com exercícios de intensidade moderada (40-60% da capacidade máxima) durante oito semanas em ratas ovariectomizadas com diabetes induzido por STZ. Os autores demonstraram que um aumento em 66% na capacidade de corrida, em comparação ao grupo DM não treinado, preveniu a redução na fração de encurtamento e na velocidade do encurtamento da fibra, avaliadas por ecocardiografia. Quinteiro et al., ^11^ utilizando um protocolo de esteira de intensidade baixa a moderada (40-60% da velocidade máxima de corrida) durante oito semanas em ratas ovariectomizadas com diabetes induzido por STZ, observaram uma melhora de 74% na capacidade do exercício, em comparação ao grupo DM não treinado, o que também foi associado com melhora na fração de encurtamento e na velocidade de encurtamento da fibra. No presente estudo, o DM-HIIT melhorou em 57,7% a capacidade do exercício em comparação ao grupo DM-NT, enquanto essa melhora foi de apenas 21,4% no grupo DM-TC. Tal fato pode explicar a atenuação na função contrátil observada somente no grupo DM-HIIT. Ainda, nossos dados corroboram achados prévios apresentados por Khakdan et al., ^13^ que usaram a ecocardiografia para comparar a função sistólica em ratos diabéticos submetidos a oito semanas de TC (65% VO _2max_ ) ou HIIT (90%/40% VO _2max_ ) em uma esteira motorizada. O grupo HIIT melhorou a fração de ejeção e a fração de encurtamento em comparação aos valores pré-exercício, o que não foi observado no grupo TC. De acordo com o autor, o HIIT promoveu diminuição da expressão de miR-195 e microRNA, envolvidos na cardiopatia diabética.

Os efeitos do exercício sobre a contratilidade poderiam também estar relacionados com a atenuação de anormalidades no manejo do cálcio intracelular, o que se demonstrou deficiente no DM. ^31^ No coração diabético, redução na expressão e na atividade da SERCA, ^32^ na saída de Ca ^2^ através da RyR2, ^33^ e na expressão e atividade de NCX ^34^ são comumente relatadas. Todas essas alterações podem levar à sobrecarga de Ca ^2^ intracelular, ^35^ e contratilidade diminuída. ^36^ O treinamento diminui os níveis de diacilglicerol (DAG) no miocárdio, ^37^ normaliza a atividade de CaMKII, e atenua a fosforilação de RyR2 e vazamento de Ca ^2^ , atenuando a disfunção cardíaca.

Dada a escassez de dados investigando os efeitos de HIIT sobre o controle autonômico na população diabética, nós investigamos o impacto dessa modalidade de exercício sobre a VFC. ^38^ No entanto, nem o protocolo HIIT de curto prazo nem o TC prejudicou ou promoveu melhoras nos parâmetros de VFC em animais diabéticos durante o período avaliado. Tal fato demonstra que, embora uma maior demanda e uma maior ativação da função cardíaca seja necessária durante o ciclo de alta intensidade no HIIT, não houve um impacto negativo em curto prazo sobre a VFC, uma ferramenta para avaliação do controle autonômico. Dados de um ensaio clínico recente ^39^ demonstraram que mesmo após 12 semanas de HIIT não supervisionado em indivíduos com DM2, o exercício não afetou a VFC, corroborando nossos achados. Um protocolo de exercício de alta intensidade em uma esteira (treino não intervalado) de 10 semanas foi capaz de atenuar as alterações simpático-vagais relatadas (maior razão LF/HF) em ratos diabéticos sedentários em um modelo de hiperglicemia moderada. ^40^

Índices deficientes da VFC global, tais como SDNN e potência total foram observados 70 dias ^41^ e 80 dias ^42^ após a indução de DM por STZ. No segundo caso, ^42^ os índices deficientes da VFC global foram associados com uma redução na FC e na razão LF/HF. No entanto, Howarth et al., ^43^ relataram, em ratos após quatro semanas de tratamento com STZ, redução na FC e aumento na razão LF/HF. Por outro lado, nossos dados não demonstraram reduções na VFC 35 dias após o tratamento com STZ, o que sugere a possibilidade de nosso protocolo ter sido muito curto para induzir NAC.

Observamos, em animais diabéticos treinados, uma redução significativa na FC em comparação a seus respectivos controles no final do protocolo de exercício. Isso também foi observado na FC espontânea de corações isolados perfundidos de todos os grupos DM ( Figura 2C ). Diminuições na FC após a administração de STZ foram são comumente relatadas. ^44^ Tal fato pode ser, em parte, atribuído a um efeito tóxico da STZ no coração, ^45^ que pode levar à disfunção do nó sinoatrial. ^44 , 46^ A corrente *funny* (I _f_ ), carregada por canais controlados por nucleotídeos cíclicos ativados por hiperpolarização (HCN) exerce função importante no controle da FC. ^47^ Nas células do nó sinoatrial, a isoforma do HCN mais abundante é o HCN4, seguido de HCN2. ^48^ Baruscotti et al., ^47^ mostraram em camundongos *knock-out* para HCN4 uma redução de 70% na corrente I _f,_ 50% na FC e 60% na FC espontânea. Redução na expressão dos canais HCN e sua proporção pode ser a razão da disfunção na FC basal. No nódulo sinoatrial dos ratos com diabetes induzido por STZ, Huang et al., ^44^ demonstraram uma diminuição da expressão de 70% de HCN2 e 58% de HCN4, e Kondo et al., ^46^ relataram uma redução na expressão de HCN4 atribuída a um número reduzido de células no nódulo sinoatrial. Silva et al., ^49^ usando ivabradina, um bloqueador de HCN4, mostram reduções na FC sem alterações no controle autonômico cardíaco. Tal fato pode explicar a redução na FC sem impacto sobre a VFC durante o período de estudo.

Nosso estudo teve algumas limitações, como a curta duração do protocolo de intervenção e do acompanhamento, o qual não foi longo o suficiente para se detectar mudanças na VFC em animais diabéticos. Assim, estudos futuros são necessários para investigar os efeitos dos mesmos protocolos de exercícios em animais com DM e NAC. Ainda, nós conseguimos avaliar a função cardíaca em corações isolados somente em um momento. Uma avaliação repetida não invasiva, como por ecocardiografia, poderia determinar mudanças progressivas ocasionadas pelo HIIT na função cardíaca.

## Conclusões

HIIT de curto prazo promove maior melhora no desempenho no exercício em comparação ao TC, inclusive no DM, sem alterar de maneira significativa a VFC.
