# Supplementary material for: Efeitos do Treinamento Intervalado de Alta Intensidade e do Treinamento Contínuo na Capacidade de Exercício, Variabilidade da Frequência Cardíaca e em Corações Isolados em Ratos Diabéticos
Source: Arq Bras Cardiol. 2022 Dec 20;120(1):e20220396. [Article in Portuguese] doi: 10.36660/abc.20220396 (PMC9833297; doi:10.36660/abc.20220396)
Supplement: Translation

# Translation: Effects of High-Intensity Interval Training and Continuous Training on Exercise Capacity, Heart Rate Variability and Isolated Hearts in Diabetic Rats

**Authors:** Eduardo Gomes de Souza, João Victor Capelli Peixoto, Claucio Rank, Ricardo Rasmussen Petterle, Rosalvo Tadeu Hochmuller Fogaça, Beata Maria Wolska, Fernando Augusto Lavezzo Dias

**Affiliations:** 1 Brazil Universidade Federal do Paraná – Departamento de Fisiologia, Curitiba, PR – Brazil; 2 Brazil Universidade Federal do Paraná – Departamento de Medicina Integrada, Curitiba, PR – Brazil; 3 USA University of Illinois at Chicago – Medicine, Physiology and Biophysics, Chicago, Illinois – USA

**Keywords:** Exercise, Physical Exertion, Diabetes Mellitus, Rats, Heart Rate

## Abstract

**Background:**

High-intensity interval training (HIIT) has been suggested as an alternative for continuous training (CT) in people with diabetes mellitus (DM) due to its short duration and potential to improve adherence to exercise. However, data on its impact on heart rate variability (HRV) are scarce.

**Objectives:**

To assess and compare the effects of HIIT and CT on exercise capacity, HRV and isolated hearts in diabetic rats.

**Methods:**

DM (intravenous streptozotocin, 45 mg.kg^-1^) and control (C) animals performed 20 sessions (5 days/week, 50 min, for 4 weeks) of CT on a treadmill (70% of maximal exercise capacity) or HIIT (cycles of 1:1min at 50% and 90% of maximal exercise capacity). HRV was assessed by continuous electrocardiogram, and cardiac function assessed in isolated perfused hearts. For data analysis, we used the framework of the multivariate covariance generalized linear model or one-way ANOVA followed by Tukey’s test, considering p<0.05 as significant.

**Results:**

Higher exercise capacity (m/min) was achieved in HIIT (DM-HIIT: 36.5 [IQR 30.0-41.3]; C-HIIT: 41.5 [37.8-44.5], both n=10) compared to CT (DM-CT: 29.0 [23.8-33.0]; C-CT: 32.0 [29.5-37.0], both n=10) (p<0.001). Heart rate (bpm) was lower in DM compared to controls (p<0.001) both in vivo (DM-HIIT:348±51, C-HIIT:441±66, DM-CT:361±70, C-CT:437±38) and in isolated hearts. There were no differences in HRV between the groups. Maximum and minimal dP/dt were reduced in DM, except +dP/dt in DM-HIIT vs. C-HIIT (mean difference: 595.5±250.3, p=0.190).

**Conclusion:**

Short-term HIIT promotes greater improvement in exercise performance compared to CT, including in DM, without causing significant changes in HRV.

## Introduction

Diabetes mellitus (DM) is a risk factor for cardiovascular disease and is associated with higher all-cause and cardiovascular mortality.^1^Among the well-known DM complications is neuropathy.^2^

Cardiovascular autonomic neuropathy (CAN), frequently observed in people with DM,^3^affects the autonomic fibers that innervate the heart and blood vessels.^4^Impaired nerve function causes physiological changes as increased heart rate (HR) and reduced heart rate variability (HRV) at rest in these individuals.^5^In addition to DM patients, reductions in HRV are also reported in diseases related to sedentary lifestyle such as hypertension,^6^obesity,^7^and myocardial infarction.^8^Therefore, the regular practice of physical exercise has become an important tool in health promotion.^9^

Diabetic animals present reduced physical capacity when compared to controls.^10 , 11^In the heart, this condition reflects negatively on systolic^10 - 12^and diastolic function^10^and is also associated with cardiac overload.^10^Physical training restores the functional capacity of diabetic animals which is accompanied by improvement in cardiac dysfunction.^10 , 11 , 13^However, the magnitude of the improvement is dependent on exercise intensity and may depend on the modality of the exercise training.^13 , 14^

High-intensity interval training (HIIT) has been considered an effective way to increase regularity in performing physical exercise due to short duration^15^and superior adaptive responses when compared to continuous training (CT). Thus, HIIT has been suggested for people with diabetes,^16 - 18^even without an in-depth knowledge of the physiological repercussions on autonomic cardiac control in this population.^19^Clear evidence of the safety of HIIT, and its beneficial effects on the cardiovascular system is necessary for its recommendation for DM patients.

We hypothesized that compared to CT, HIIT program improves exercise capacity but may have a different effect on HRV and cardiac contractility in a model of streptozotocin (STZ)-induced diabetes. Therefore, the objective of this study was to evaluate, in STZ-induced diabetic rats, the effects of short-term protocol of HIIT or CT on exercise capacity, HRV assessed by electrocardiogram (EKG), and on cardiac function in isolated perfused heart.

## Methods

### Animals and experimental design

Sixty male Wistar rats, weighing 250-300 g were kept in cages under controlled conditions of temperature and a light-dark cycle of 12 h, with free access to food and water and randomly divided in control (C, n=30) and DM groups (DM, n=30). These groups were subdivided in non-trained control (C-NT, n=10), non-trained *diabetes* (DM-NT, n=10), CT control (C-CT, n=10), DM animals in CT (DM-CT, n=10), interval training control (C-HIIT, n=10) and interval training *diabetes* (DM-HIIT, n=10). All experimental protocols used in this study were approved by the Ethics Committee Animal Experimentation of the Division of Biological Sciences of the Federal University of Paraná (approval number: AEEC-866) and conducted in accordance with the National Council on Animal Experimentation (CONCEA) guidelines.

Initial sample size was calculated using GPower 3.1 based on percentage increase of exercise capacity observed in previous experiments. Six independent groups of equal number (n), effect size of 0.6, power of 0.85, and alpha of 0.05, were considered, resulting in a sample size of 48 animals (eight per group).

The experimental design is represented in Figure 1 and described in detail below.

**Figure 1:** Schematic diagram of the experimental design; EKG: electrocardiogram; DM: diabetes mellitus.

### Electrode implantation and DM induction

After 14 hours of fasting, all animals were anesthetized with ketamine (80 mg kg^-1^, Ketalex, Dechra, Brazil) and xylazine (10 mg kg^-1^, Xilazin, Syntec, Brazil) for the procedure. Four electrodes (stainless steel, 0.5mm) were subcutaneously implanted posterior to each limb as described by Marques Neto et al.^20^for EKG monitoring. DM was subsequently induced by an intravenous injection (penile vein) of STZ (45 mg.kg^-1^, Sigma-Aldrich, Germany) solubilized in 0.01M of citrate buffer, pH 4.5.^21^In the control group, only citrate buffer was injected. For capillary glycemia (preceded from six-hour fasting) blood samples were collected from the rats’ tail vein and measured using a digital glucometer (Accu-check Performa, Roche Diagnostic, Germany) before the STZ injection, seven days after the injection to confirm the hyperglycemic state (fasting blood glucose >250 mg dL^-1^), and at the beginning, on the 15^th^day and at the end of the training protocol.

### EKG monitoring and HRV calculation

Animals were kept in individual cages and EKG was recorded 24 hours after DM confirmation, before the exercise performance test (described below), and repeated once a week during the four weeks of the exercise protocol. Measurements were performed for one hour, always in the morning to avoid the influence of circadian cycle,^22^and without restriction or anesthesia (cables were attached to the implanted wires but animals were able to move inside the cages). Experiments were performed in a silent and reserved room, with a temperature of 20^o^C.

Frontal plane EKG was acquired using a PowerLab, model 26T (AD Instrument, Australia) acquisition system and subsequently analyzed using Lab Chart version 7.0 software (AD Instrument, Australia). For HRV, data obtained from DII lead was processed in LabChart and later transferred to Kubius HRV (2.0 MATLAB, MathWorks, Inc., Finland) for analysis.

HRV was measured by acquiring RR intervals within one millisecond resolution. Data were analyzed in the time and frequency domains using the area of greatest stability in RR intervals corresponding to 10 minutes of recordings. For the time domain parameters, we calculated the normal-to-normal intervals (SDNN), the square root of the mean of the squares of successive RR interval differences (rMSSD), and HR. Frequency domains were analyzed by fast Fourier transform after subtracting the linear tendency using automatic filters. We then calculated low frequency (LF) (0.20 to 0.75 Hz), high frequency (HF) (0.75 to 3Hz) and the LF/HF ratio. Furthermore, non-linear analysis for SD1 (standard deviation of the instantaneous RR variability) and SD2 (standard deviation of the continuous or long term variability of the heart rate) was performed.^23^

### Exercise performance test and training protocol

Exercise training was performed in a motorized treadmill designed for rodents (Insight Equipamentos Ltda, Brazil) with speed controlled by computer. The exercise performance test and the training protocol were adapted from Pereira et al.^24^

Twenty-four hours after DM confirmation, the animals started to be adapted to run on the treadmill. The adaptation was performed on five consecutive days, 50 minutes per day, without inclination. On the first day, the animals walked at 5 m/min. The speed was increased by 1 m/min per day. On the sixth day, the animals were submitted to an exhaustion protocol aiming at identifying the maximum running speed of each animal. The test consisted of graded exercise (no incline) starting at 5 m/min with increments of 1 m/min every one minute, up to the maximal running speed attained by each rat (exhaustion). When the animal could not maintain the speed for a minute, the previous speed they were able to run was considered as the maximum attained speed.

The physical exercise training sessions started 48 hours after the test and were performed five times a week (Monday to Friday) for four weeks, 50 minutes per session. All sessions started with a 5-minute warm-up and ended with a 5-minute cool down at 5 m/min. The CT groups ran at 70% of the individual maximum capacity for 40 minutes. The interval training groups ran at 90% of the individual maximum capacity for one minute interspersed with one minute at 50% for 40 minutes. Non-trained groups remained on the treadmills for 50 minutes without exercising. After 48 hours from the last training session, the animals were resubmitted to the exercise performance test, as described above.

### Cardiac function in isolated perfused heart

Cardiac function was assessed in isolated perfused hearts as described before.^25^Briefly, animals were weighed, anesthetized with an intraperitoneal injection of ketamine (80 mg kg^-1^) and xylazine (20 mg kg^-1^), and euthanized by exsanguination. The chest was opened, the heart collected and quickly transferred to a Langendorff perfusion system, allowing immediate perfusion with Krebs-Henseleit’s solution. This solution had the following composition (in mM): NaCl = 118.5, KCl = 5, CaCl_2_= 1.5, KH_2_PO_4_= 2.0, MgSO_4_= 1.2, NaHCO_3_= 26 and glucose = 10, maintained at pH 7.4 and gassed with carbogen (95% O_2_/5% CO_2_) at 37°C and constant perfusion pressure of 60 mmHg. For measurement of the left ventricular pressure, a portion of the left atrium was removed, and a plastic balloon connected to a pressure transducer (WPI-rBPI, USA) was inserted into the left ventricle through the mitral valve. The volume of the balloon was gradually adjusted to obtain the maximum developed pressure in spontaneous beating hearts. The data were obtained using an acquisition system PowerLab 26T and analyzed using Lab Chart version 7.3.7 software (AD Instruments, Australia).

### Statistical analysis

Results are expressed as mean values and standard deviation of the mean or median and interquartile range for non-parametric data. For data analysis, we used the framework of the multivariate covariance generalized linear model^26^through McGLM package^27^with appropriate correction for sample distribution using Tweedie test (using logarithmic link function for the linear predictor). We used the Shapiro-Wilk test to assess whether the variables followed a normal distribution. For data of isolated heart, since we only have one time-point, we used a one-way ANOVA and Tukey’s test for multiple comparisons.

For data analysis and plotting we used R software (The R foundation, https://www.r-project.org/), or plotting using Graph Pad Prism 8 (Graph Pad Software, San Diego, California, USA). The accepted level of significance was p < 0.05.

## Results

### Body weight, glycemia and exercise capacity

Body weight, glucose levels, and exercise capacity before and after the exercise protocol in control and diabetic animals are represented in Table 1 . No differences were observed for weight between the groups (p=0.877) at baseline. Weight decreased in DM animals (estimate: -19.1, p<0.001) and increased in controls (estimate: 66.57, p<0.001) during the follow-up. Body weight was significantly lower in diabetic animals compared to controls after the exercise protocol period (p<0.001). There was no effect of exercise modality on body weight change after the follow-up period (p=0.91 comparing exercise modalities).

**Table 1:** Body weight, glycemia and exercise capacity in control and diabetic animals before and after the exercise protocol

	C-NT (n=10)	DM-NT (n=10)	C-CT (n=10)	DM-CT (n=10)	C-HIIT (n=10)	DM-HIIT (n=10)
**Body weight (g)**						
Before	266.50 ± 8.41	281.00 ± 22.53	261.00 ± 26.23	250.80 ± 16.71	261.10 ± 22.61	259.80± 19.27
After	342.60 ± 13.18^a^	257.40 ± 33.27^a b^	320.30 ± 31.71^a^	247.00 ± 44.22^a b^	325.40 ± 16.31^a^	229.90 ± 24.28^a b^
**Blood glucose (mg/dL** ^**-1**^ **)**						
Before	94.60 ± 10.33	462.90 ± 61.21^b^	102.50 ± 3.69	450.00 ± 143.5^b^	106.30 ± 10.33	489.60 ± 72.22^b^
After	94.30 ± 5.48	504.20 ± 66.55^a b^	104.10 ± 7.31	542.00 ± 60.60^a b^	106.00 ± 8.97	521.90 ± 64.08^a b^
**Maximum running speed (m/min)**						
Before	27.00 (25.75-29.25)	25.5 (23.00-27.25)	25.00 (24.50-28.00)	24.50 (23.25-25.00)	25.00 (24.50-31.00)	24.50 (20.75-25.00)
After	25.00(23.75-25.50)^a^	21.50 (19.00-25.25)^a^	32.00 (29.50-37.00)^ac^	29.00 (23.75-33.00)^ac^	41.50 (37.75-44.50)^acd^	36.50 (30.00-41.25)^acd^

*Data are expressed as mean ± SD or median (interquartile range). a Statistically significantly different (p<0.001) when compared to before (baseline); b statistically significantly different (p<0.001) when compared to controls; c statistically significantly different (p<0.001) when compared with NT; d statistically significantly different (p<0.001) when compared to CT; C-NT: non-trained controls, DM-NT: non-trained diabetes; controls in continuous training; DM-CT: diabetic animals in continuous training; C-HIIT: control animals in high-intensity interval training; DM-HIIT : diabetic animals in high-intensity interval training*

Glucose levels were higher in DM compared to controls at baseline (estimate: 366.4mg/dL, p<0.001) and at follow-up (estimate: 421.2mg/dL, p<0.001). The follow-up glucose levels further increased in DM animals but not in controls (p=0.982). There was no significant effect of exercise on blood glucose levels ( Table 1 ).

Maximum running speed was not different between the groups at baseline. Non-trained animals demonstrated a significant decrease in maximum running speed at follow-up. Although both exercise modalities improved maximum exercise speed, HIIT promoted higher exercise performance as compared to CT. Mean maximum running speed was increased by 27.3% in C-CT and 21.4% for DM-CT. Animals submitted to interval training had their exercise capacity increased by an average 52.9% for C-HIIT and 57.7% for DM-HIIT. There was no difference between DM and controls in the variation in maximum running speed promoted by the exercise protocol at follow-up (no statistical significance when triple interaction was assessed).

### Time and frequency domains, and nonlinear measures of HRV

Parameters of HR and HRV during the experimental period are summarized in Table 2 . There were no differences in HRV parameters related to global variability or sympathovagal balance between the groups before or after the exercise protocol. However, a reduction in heart rate between DM animals and controls was observed.

**Table 2:** Heart rate and heart rate variability parameter in control and diabetic rats before and after the exercise protocol

	C-NT	DM-NT	C-CT	DM-CT	C-HIIT	DM-HIIT
**Heart rate (bpm)**						
Before	404.15 ± 49.33	358.15 ± 41.51^b^	405.62 ± 42.30	372.06 ± 50.80^b^	411.47 ± 32.37	339.50 ± 50.52^b^
After	405.74 ± 36.48	342.69 ± 43.89^b^	436.74 ± 38.21	361.18 ± 70.72^b^	441.26 ± 66.60	347.80 ± 50.56^b^
**SDNN (ms)**						
Before	7.20 (5.84-10.14)	10.25 (6.26-15.03)	6.37 (4.51-8.08)	8.33 (5.81-13.61)	10.69 (6.44-11.84)	9.11 (6.91-11.22)
After	6.20 (5.440-9.00)	11.54 (6.59-13.92)	8.30 (5.90-11.83)	10.49 (7.00-13.91)	8.52 (5.86-12.08)	7.94 (6.14-10.03)
**RMSSD (ms)**						
Before	3.56 (2.37-3.66)	2.57 (2.15-3.64)	3.13 (2.16-4.05)	3.25 (2.29-3.54)	3.01 (2.56-4.20)	3.13 (2.20-5.72)
After	3.06 (2.26-4.05)	4.21 (2.41-5.20)	2.91 (2.02-4.41)	3.44 (2.59-4.26)	2.80 (1.89-3.765)	2.67 (1.90-3.64)
**Total power (ms** ^**2**^ **)**						
Before	36.02 (26.69-59.07)	66.97 (16.97-129.4)	32.99 (14.93-59.02)	38.62 (32.32-103.8)	62.89 (31.87-92.58)	58.05 (22.33-81.79)
After	33.91 (20.56-64.02)	83.56 (34.71-115.4)	65.14 (25.50-138.9)	79.64 (32.93-143.3)	60.77 (17.32-117.6)	42.32 (27.22-98.79)
**LF (ms** ^**2**^ **)**						
Before	3.63 (1.42-4.98)	1.93 (1.26-5.32)	2.31 (1.13-8.68)	2.20 (1.90-3.76)	2.45 (1.95-11.4)	3.40 (1.29-12.47)
After	2.62 (1.51-7.91)	5.88 (1.72-13.86)	2.37 (0.71-15.36)	2.78 (1.84-6.57)	3.09 (1.41-7.37)	3.25 (1.65-5.17)
**HF (ms** ^**2**^ **)**						
Before	5.43 (3.45-6.33)	2.70 (1.16-7.79)	4.01 (1.44-9.31)	5.36 (2.08-7.59)	4.92 (3.06-9.81)	5.49 (1.44-9.31)
After	4.02 (1.82-8.02)	6.23 (2.91-10.29)	4.03 (1.57-8.40)	4.12 (2.57-6.91)	4.33 (0.99-7.85)	1.87 (1.20-4.81)
**LF/HF**						
Before	0.79 (0.56-1.04)	0.78 (0.47-1.41)	0.55 (0.44-1.53)	0.74 (0.47-0.98)	0.66 (0.52-1.13)	0.91 (0.65-1.18)
After	0.78 (0.57-1.44)	0.95 (0.59-1.46)	0.69 (0.40-1.42)	0.77 (0.43-1.04)	0.70 (0.49-1.00)	1.25 (0.79-1.75)
**SD1**						
Before	2.52 (1.68-2.59)	1.82 (1.52-2.58)	2.21 (1.53-2.86)	2.30 (1.62-2.50)	2.13 (1.81-2.97)	2.46 (1.56-4.04)
After	2.16 (1.60-2.86)	2.98 (1.71-3.67)	2.06 (1.43-3.12)	2.44 (1.83-3.02)	1.98 (1.34-2.66)	1.89 (1.33-2.57)
**SD2**						
Before	9.94 (8.08-13.98)	14.29 (8.70-21.05)	8.49 (6.02-11.04)	11.59 (7.97-19.16)	14.80 (8.81-16.52)	12.17 (9.58-15.57)
After	8.57 (7.42-12.39)	16.04 (9.24-19.41)	11.68 (8.06-16.43)	14.68 (9.58-19.32)	11.77 (8.07-16.91)	10.90 (8.53-14.06)

*Data are present as mean ± SD or median (interquartile range). SDNN: Standard deviation of all R-R intervals. RMSSD: Square root of the mean of the squares of successive R-R interval differences. LF: low frequency. HF: high frequency. SD1: standard deviation of the instantaneous RR variability. SD2 standard deviation of the continuous or long-term variability of the heart rate.^*b*^Represents statistical difference when DM is compared to Control. N is equal 10 except for HRV parameter in C-CT (n=9); C-NT: non-trained controls, DM-NT: non-trained diabetes; controls in continuous training; DM-CT: diabetic animals in continuous training; C-HIIT: control animals in high-intensity interval training; DM-HIIT: diabetic animals in high-intensity interval training.*

### Isolated perfused heart

Left ventricular developed pressure (LVDP) is represented in Figure 2 . Control animals submitted to exercise differed from the DM-NT, C-HIIT, and DM-CT groups; however, there was no differences between DM and C submitted to same conditions (i.e., NT or different types of training; p = 0.086 for NT animals, p = 0.061 for CT and p = 0.824 for HIIT).

**Figure 2:** Heart rate, left ventricular developed pressure, and maximum rate of left ventricular pressure rise and pressure fall in isolated perfused hearts. A: Left ventricular developed pressure (LVDP). B: Maximum rate of left ventricular pressure rise (+dP/dt) and fall (–dP/dt). C: Heart rate (HR). *Statistically significant difference (p<0.01) compared to DM-NT, # statistically significant (p<0.05) compared to DM-CT, § statistically significant (p<0.05) compared to all DM groups.

Significant differences were observed in +dP/dt (mmHg/s) in DM groups compared with respective controls (C-NT vs. DM-NT, mean difference: 812.8 ± 228.5, p = 0.012; C-CT vs. DM-CT, mean difference: 937.5 ± 207.7, p = 0.0008) except for HIIT (C-HIIT vs. DM-HIIT, mean difference: 595.5 ± 250.3, p = 0.190) ( Figure 2B ). Lower values were also observed for –dP/dt ( Figure 2B ) in all DM groups compared with controls. Spontaneous HR ( Figure 2C ) was reduced in all DM groups compared with controls (C-NT vs. DM-NT, mean difference: 31.08 ± 8.48; C-CT vs. DM-CT, mean difference: 34.23 ± 7.71; C-HIIT vs. DM-HIIT, mean difference: 42.58 ± 9.29).

## Discussion

This study compared, in STZ-induced diabetic rats, the effects of 20 sessions of HIIT or CT on exercise capacity, cardiac function and HRV. Our results demonstrated that both types of training improved exercise performance, with better results with HIIT, and comparable magnitudes between DM and controls animals. Furthermore, there were no differences in HRV between controls and DM after the exercise protocols. HIIT attenuated the impairment in +dP/dt, but not –dP/dt in isolated hearts from DM.

Diabetic animals submitted to training increased their exercise performance ( Table 1 ), as opposed to sedentary animals whose maximum running speed was significantly reduced. Nevertheless, DM and control animals submitted to HIIT showed greater improvement in maximum running speed when compared with animals submitted to CT, even though glucose levels did not change. The superiority of interval training over CT had been previously described in a rat model of metabolic syndrome, where VO_2max_was superior and associated with a greater reduction in blood pressure, better endothelial function, and superior insulin action.^14^In a mouse model of diabetes, HIIT did reduce fasting glucose and increased muscle GLUT4 content; however, this study protocol was performed for 10 weeks compared to four weeks in our study, which may explain the difference in glucose levels.^28^Furthermore, in humans, Driller et al.^29^showed improvement in power, maximum oxygen uptake, and lactate threshold in rowers submitted to HIIT as compared to traditional training. In type 2 diabetic patients, a recent systematic review described a higher increase in VO_2max_in HIIT *versus* CT, without significant differences in HbA1c or blood pressure values between interventions, similar to our findings.^30^The greater improvement in physical capacity found in our study using a short-term HIIT is in accordance with adaptive responses promoted by HIIT protocols previously reported.

Cardiac function analysis in isolated hearts revealed no statistical differences in left ventricular developed pressure in spontaneously beating hearts between groups submitted to the same exercise protocol ( Figure 2A ). However, DM-HIIT showed attenuation in +dP/dt impairment, which was not observed in DM-CT ( Figure 2B ). No improvement was observed in diastolic function, represented by –dP/dt, in any of DM exercised groups ( Figure 2B ). Previous reports correlated increased exercise capacity with improved systolic function in DM. Sanches et al.^10^used a combined protocol of aerobic (treadmill) and resistance training (ladder) with low to moderate intensity (40-60% of maximal capacity) during eight weeks in ovariectomized STZ-induced diabetic rats. The authors demonstrated that an improvement of 66% in running capacity, compared to non-trained DM group, prevented reduction in fractional shortening and velocity of fiber shortening, assessed by echocardiography. Quinteiro et al.,^11^using a low to moderate (40-60% of maximal running speed) treadmill protocol during eight weeks in ovariectomized STZ-induced rats observed an improvement of 74% in the exercise capacity, when compared to non-trained DM group that was also associated with improvement in fractional shortening and fiber shortening velocity. In the present study, DM-HIIT showed a 57.7% improvement in exercise capacity when compared to DM-NT while the DM-CT showed an improvement of only 21.4%. This may explain the attenuation in contractile function observed only in the DM-HIIT group. Also, our data corroborates with previous findings by Khakdan et al.,^13^that used echocardiography to compare the systolic function in DM rats submitted to eight weeks of continuous training (65% VO_2max_) or HIIT (90%/40% VO_2max_) in a motorized treadmill. HIIT group improved ejection fraction and fractional shortening when compared to pre-exercise values, which was not observed in the CT group. According to the author, HIIT decreased the expression of miR-195, microRNA involved in diabetic cardiopathy.

The effects of exercise training on contractility could also be related to attenuation in abnormalities of intracellular Ca^2^handling, that has been shown to be impaired in DM.^31^In diabetic heart, reduced expression and activity of SERCA2,^32^Ca^2^leak thru RyR2^33^and decreased NCX expression and activity^34^are commonly reported. All these changes may lead to intracellular Ca^2^overload,^35^and decreased contractility.^36^Exercise training decreases myocardial diacylglycerol (DAG) levels,^37^normalizes CaMKII activity and attenuates RyR2 phosphorylation and Ca^2^leakage, attenuating cardiac dysfunction.

Due to the scarcity of data investigating the effects of HIIT on autonomic control in the diabetic population we investigated the impact of this exercise modality on HRV.^38^The high physical effort during peak exercise in HIIT could be thought of as a stressor for the already challenged cardiovascular autonomic control in the diabetic. However, neither short-term protocol of HIIT or CT impaired or caused improvements in HRV parameters in DM animals during the period assessed. This demonstrates that, even though higher cardiac demand and activation is required during high-intensity cycle in HIIT training, there was no short-term negative impact on HRV, a tool for autonomic control assessment. Data from a recent clinical trial^39^demonstrated that even after 12 weeks of unsupervised HIIT in DM2 subjects, there were no effects of exercise on HRV, corroborating our findings. A high-intensity exercise protocol in a treadmill (not interval training) of 10 weeks was able to attenuate the sympathovagal changes (higher LF/HF ratio) in sedentary DM rats in a model of moderate hyperglycemia.^40^

Impaired indexes of global HRV such as SDNN and total power have been previously observed 70 days^41^and 80 days^42^after STZ-induced DM. In the latter case^42^impaired indexes of global HRV were associated with decrements in HF and LF/HF ratio. However, Howarth et al.^43^reported in rats after four weeks of STZ-treatment, decrements in HF and increments in the LF/HF ratio. On the other hand, our data did not show any decrements in HRV after 35 days from STZ-treatment, which may suggest that our protocol was too short to induce CAN.

We observed in DM-trained animals a significant decrement in HR compared to their respective controls at the end of the exercise protocol. This was also observed in spontaneous HR of isolated perfused hearts for all DM groups ( Figure 2C ). HR decrements after STZ administration are commonly reported.^44^This may be in part attributed to a toxic effect of STZ in the heart^45^that may lead to sinoatrial node dysfunction.^44 , 46^The pacemaker current (I_f_) carried by the hyperpolarization-activated cyclic nucleotide-gated (HCN) channels plays an important role in HR control.^47^In the sinoatrial node cells, the most abundant HCN isoform is HCN4 followed by HCN2.^48^Baruscotti et al.^47^showed in HCN4 knock-out mice a decrement of 70% in I_f_current, 50% in HR and 60% in spontaneous HR. Reduction in the expression of HCN channels and their proportion may be the reason for the impaired basal HR. In sinoatrial node of STZ-induced diabetic rats, Huang et al.^44^demonstrated a decrease in the expression of 70% in HCN2 and 58% in HCN4 and Kondo et al.^46^reported a decrease in HCN4 expression attributed to reduced number of sinoatrial node cells. Silva et al.,^49^using ivabradine, an HCN4 blocker, demonstrated decrements in HR without changes in cardiac autonomic control. This may be the reason why we observed reduced HR without impact on HRV during the observed period.

Our study had some limitations, such as the short duration of the intervention protocol and follow-up that was not long enough to detect changes in HRV in DM animals. Therefore, future studies are warranted to investigate the same exercise protocols in DM animals with established CAN. In addition, we were only able to assess cardiac function in one time-point using isolated hearts. A repetitive and non-invasive assessment, such as echocardiography, could determine progressive changes induced by HIIT in cardiac function.

## Conclusions

Short-term HIIT promotes greater improvement in exercise performance compared to CT, including in DM, without causing significant changes in HRV.
